# Lyapunov-type inequalities for mixed non-linear forced differential equations within conformable derivatives

**DOI:** 10.1186/s13660-018-1731-x

**Published:** 2018-06-20

**Authors:** Thabet Abdeljawad, Ravi P. Agarwal, Jehad Alzabut, Fahd Jarad, Abdullah Özbekler

**Affiliations:** 1 Department of Mathematics and General Sciences, Prince Sultan University, Riyadh, Saudi Arabia; 2 Department of Mathematics, Texas A&M University–Kingsville, Kingsville, USA; 3 Department of Mathematics, Çankaya University, Ankara, Turkey; 4 Department of Mathematics, Atilim University, Ankara, Turkey

**Keywords:** 34A08, 26D15, Lyapunov inequality, Hartman inequality, Conformable derivative, Green’s function, Boundary value problem, Mixed non-linearities

## Abstract

We state and prove new generalized Lyapunov-type and Hartman-type inequalities for a conformable boundary value problem of order $\alpha \in (1,2]$ with mixed non-linearities of the form

$$ \bigl(\mathbf{T}_{\alpha }^{a} x\bigr) (t)+r_{1}(t) \bigl\vert x(t) \bigr\vert ^{\eta -1}x(t)+r_{2}(t)\bigl\vert x(t) \bigr\vert ^{ \delta -1}x(t)=g(t), \quad t\in (a,b), $$

satisfying the Dirichlet boundary conditions $x(a)=x(b)=0$, where $r_{1}$, $r_{2}$, and *g* are real-valued integrable functions, and the non-linearities satisfy the conditions $0<\eta <1<\delta <2$. Moreover, Lyapunov-type and Hartman-type inequalities are obtained when the conformable derivative $\mathbf{T}_{\alpha }^{a}$ is replaced by a sequential conformable derivative $\mathbf{T}_{\alpha }^{a} \circ \mathbf{T}_{\alpha }^{a}$, $\alpha \in (1/2,1]$. The potential functions $r_{1}$, $r_{2}$ as well as the forcing term *g* require no sign restrictions. The obtained inequalities generalize some existing results in the literature.

## Introduction

The Lyapunov inequality and its generalizations have been indispensable in the investigation of various topics of differential equations including oscillation theory, stability theory, intervals of disconjugacy, and eigenvalue problems [1–5]. The problem was initiated by Lyapunov himself who established a necessary condition for the existence of solutions for the boundary value problem (BVP)

1.1 $$\begin{aligned} \textstyle\begin{cases} x''(t)+r(t)x(t)=0,&t \in (a,b), \\ x(a)=x(b)=0. \end{cases}\displaystyle \end{aligned}$$

Indeed, he proved in [6] that if BVP (1.1) has a non-trivial solution, then the inequality

1.2 $$ \int_{a}^{b} \bigl\vert r(t) \bigr\vert \,\mathrm{d}t> \frac{4}{b-a} $$

holds, where *r* is a real-valued integrable function. Since then (1.2) is referred to as the Lyapunov inequality. In [7], Wintner was ahead and replaced $\vert r(t) \vert $ by the function $r^{+}(t)$ and obtained the following slightly different version of the Lyapunov inequality:

1.3 $$ \int_{a}^{b} r^{+}(t)\,\mathrm{d}t> \frac{4}{b-a}, $$

where $r^{+}(t)=\max \{r(t),0\}$. This inequality is considered to be the best reachable one in the sense that the constant 4 in (1.3) cannot be replaced by any other larger constant (see [6] and [8, Theorem 5.1]). In his remarkable book, Hartman [8] was beyond this estimate and obtained a generalized version as follows:

1.4 $$ \int_{a}^{b}(b-t) (t-a)r^{+}(t)\,\mathrm{d}t>b-a. $$

The study of this problem on various types of differential and difference equations over the last years has resulted in different versions of Lyapunov-type inequalities; the reader may consult the papers [9–13] and the first chapter in [14] for a complete view. In parallel to the intensive investigation tendency amongst researchers, Agarwal et al. in [15] has recently considered the mixed non-linear BVP of the form

1.5 $$\begin{aligned} \textstyle\begin{cases} x''(t)+r_{1}(t)\vert x(t) \vert ^{\eta -1}x(t)+r_{2}(t)\vert x(t) \vert ^{\delta -1}x(t)=0, \\ x(a)=x(b)=0, \end{cases}\displaystyle \end{aligned}$$

where the non-linearities satisfy

1.6 $$\begin{aligned} 0< \eta < 1< \delta < 2 \end{aligned}$$

and no sign restrictions are imposed on the real-valued integrable potential functions $r_{1}$, $r_{2}$, and obtained the following Hartman-type and Lyapunov-type inequalities.

### Theorem 1.1

(Hartman-type inequality)

*Suppose that* *a*, *b*, $b>a$ *are consecutive zeros of a non*-*trivial solution of BVP *(1.5), *then the inequality*

1.7 $$\begin{aligned} &\eta_{0} \biggl( \int_{a}^{b}(b-t) (t-a)r_{1}^{+}(t)\,\mathrm{d}t \biggr) ^{2}+\delta_{0} \biggl( \int_{a}^{b}(b-t) (t-a)r_{2}^{+}(t)\,\mathrm{d}t \biggr) ^{2} \\ &\qquad +(\eta_{0}+\delta_{0}) \biggl( \int_{a}^{b}(b-t) (t-a)r_{1}^{+}(t)\,\mathrm{d}t \biggr) \biggl( \int_{a}^{b}(b-t) (t-a)r_{2}^{+}(t)\,\mathrm{d}t \biggr) \\ &\quad >\frac{1}{4}(b-a)^{2} \end{aligned}$$

*holds*, *where*

1.8 $$\begin{aligned} \eta_{0}=(2-\eta )\eta^{\eta /(2-\eta )}2^{2/(\eta -2)} \quad \textit{and} \quad \delta_{0}=(2-\delta )\delta^{\delta /(2-\delta )}2^{2/(\delta -2)}. \end{aligned}$$

### Theorem 1.2

(Lyapunov-type inequality)

*Suppose that* *a*, *b*, $b>a$ *are consecutive zeros of a non*-*trivial solution of BVP *(1.5), *then the inequality*

1.9 $$\begin{aligned} &\eta_{0} \biggl( \int_{a}^{b} r_{1}^{+}(t)\,\mathrm{d}t \biggr) ^{2}+ \delta_{0} \biggl( \int_{a}^{b} r_{2}^{+}(t)\,\mathrm{d}t \biggr) ^{2} +( \eta_{0}+ \delta_{0}) \biggl( \int_{a}^{b} r_{1}^{+}(t)\,\mathrm{d}t \biggr) \biggl( \int_{a}^{b} r_{2}^{+}(t)\,\mathrm{d}t \biggr) \\ &\quad >\frac{4}{(b-a)^{2}} \end{aligned}$$

*holds*, *where* $\eta_{0}$ *and* $\delta_{0}$ *are defined in* (1.8).

At some earlier time in [16] and under the same conditions, the same authors considered the mixed non-linear forced BVP of the form

1.10 $$\begin{aligned} \textstyle\begin{cases} x''(t)+r_{1}(t)\vert x(t) \vert ^{\eta -1}x(t)+r_{2}(t)\vert x(t) \vert ^{\delta -1}x(t)=g(t), \\ x(a)=x(b)=0, \end{cases}\displaystyle \end{aligned}$$

where *g* is a real-valued integrable function and obtained the following Hartman-type and Lyapunov-type inequalities.

### Theorem 1.3

(Hartman-type inequality)

*Suppose that* *a*, *b*, $b>a$ *are consecutive zeros of a non*-*trivial solution of BVP* (1.10), *then the inequality*

1.11 $$\begin{aligned} & \biggl( \int_{a}^{b}(b-t) (t-a) \bigl(r_{1}^{+}+r_{2}^{+} \bigr) (t)\,\mathrm{d}t \biggr) \\ & \qquad {}\times \biggl( \int_{a}^{b}(b-t) (t-a) \bigl\{ \eta_{0}r_{1}^{+}(t)+ \delta_{0}r_{2}^{+}(t)+\bigl\vert g(t) \bigr\vert \bigr\} \,\mathrm{d}t \biggr) \\ &\quad {}> \frac{1}{4}(b-a)^{2} \end{aligned}$$

*holds*, *where* $\eta_{0}$ *and* $\delta_{0}$ *are defined in* (1.8).

### Theorem 1.4

(Lyapunov-type inequality)

*Suppose that* *a*, *b*, $b>a$ *are consecutive zeros of a non*-*trivial solution of BVP* (1.10), *then the inequality*

1.12 $$\begin{aligned} & \biggl( \int_{a}^{b}\bigl(r_{1}^{+}+r_{2}^{+} \bigr) (t)\,\mathrm{d}t \biggr) \biggl( \int_{a}^{b} \bigl\{ \eta_{0}r_{1}^{+}(t)+ \delta_{0}r_{2}^{+}(t)+\bigl\vert g(t) \bigr\vert \bigr\} \,\mathrm{d}t \biggr) >\frac{4}{(b-a)^{2}} \end{aligned}$$

*holds*, *where* $\eta_{0}$ *and* $\delta_{0}$ *are defined in* (1.8).

It is to be noted that the forcing function *g* in (1.10) requires no sign restriction as well. Furthermore, inequality (1.11) implies (1.12) because, upon applying the arithmetic–geometric mean, we get $(b-t)(t-a)\leq (b-a)^{2}/4$ for all $t\in [a,b]$. The reader can also figure out that both inequalities (1.11) and (1.12) reduce to the classical Hartman-type and Lyapunov-type inequalities as $\eta \to 1^{-}$ and $\delta \to 1^{+}$, respectively.

Fractional differential equations have proved direct evolvement into multidisciplinary subjects such as viscoelasticity, ground water flows, boundary layer theory, granular flows, dynamics of cold atoms in optical lattices, plasma turbulence, and dynamics of polymeric materials; see, for instance, [17, 18]. The development of these equations in the last years has recently led to a tremendous number of papers which have studied different qualitative topics. Amongst them is the investigation of Lyapunov inequality which was initiated by Ferreria in [19] and continued by other scholars [20–35]. On the other hand, the newly defined conformable fractional calculus was initiated in [36] and studied later on in the papers [37, 38] where many properties of conformable operators were introduced. Apart from its simple application, nevertheless, it has been realized that this topic proves to be essential and profitable in generating new types of fractional operators [39]. However, the progress in this direction is still at its earliest stage [40–43].

The objective of this paper is to state and prove new generalized Lyapunov-type and Hartman-type inequalities for a conformable boundary value problem of order $\alpha \in (1,2]$ with mixed non-linearities of the form

$$ \bigl(\mathbf{T}_{\alpha }^{a} x\bigr) (t)+r_{1}(t) \bigl\vert x(t) \bigr\vert ^{\eta -1}x(t)+r_{2}(t)\bigl\vert x(t) \bigr\vert ^{ \delta -1}x(t)=g(t), \quad t\in (a,b), $$

satisfying the Dirichlet boundary conditions $x(a)=x(b)=0$, where $r_{1}$, $r_{2}$, and *g* are real-valued integrable functions, and the non-linearities satisfy the conditions $0<\eta <1<\delta <2$. Moreover, Lyapunov-type and Hartman-type inequalities are obtained when the conformable derivative $\mathbf{T}_{\alpha }^{a}$ is replaced by a sequential conformable derivative $\mathbf{T}_{\alpha }^{a} \circ \mathbf{T}_{\alpha }^{a}$, $\alpha \in (1/2,1]$. The potential functions $r_{1}$, $r_{2}$ as well as the forcing term *g* require no sign restrictions. The obtained inequalities generalize and compliment some existing results in the literature.

## Preliminaries on conformable derivatives

This section is devoted to stating some preliminaries on higher-order fractional conformable derivatives. We borrow the notations and terminology from the recent papers [36, 37].

### Definition 2.1

([36, 37])

The (left) conformable fractional derivative starting from *a* of a function $f:[a,\infty )\rightarrow \mathbb{R}$ of order $0<\alpha \leq 1$ is defined by

2.1 $$ \bigl(T_{\alpha }^{a} g\bigr) (t)= \lim _{\epsilon \rightarrow 0} \frac{g(t+ \epsilon (t-a)^{1-\alpha } )-g(t)}{\epsilon }. $$

In case $a=0$, we write $T_{\alpha }$. If $(T_{\alpha }^{a} g)(t)$ exists on $(a,b)$, then

$$ \bigl(T_{\alpha }^{a} g\bigr) (a)=\lim_{t \rightarrow a^{+}} \bigl(T_{\alpha }^{a} g\bigr) (t). $$

If *g* is differentiable, then one should note the following essential identity:

2.2 $$ \bigl(T_{\alpha }^{a} g\bigr) (t)=(t-a)^{1-\alpha } g^{\prime }(t). $$

Moreover, the conformable fractional integral of order $0<\alpha \leq 1$ starting at $a\geq 0$ is defined by

2.3 $$ \bigl(I_{\alpha }^{a} g\bigr) (t)= \int_{a}^{t}g(x) (x-a)^{\alpha -1}\,\mathrm{d}x $$

or following the notation in [36] as

$$ \bigl(I_{\alpha }^{a} g\bigr) (t)= \int_{a}^{t}g(x)x^{\alpha -1}\,\mathrm{d}x. $$

Throughout this article, we shall apply the conformable integral in (2.3). In case of higher order, the following definition is adopted.

### Definition 2.2

([37])

Let $n<\alpha \leq n+1 $ and set $\gamma =\alpha -n$. Then the conformable fractional derivative starting from *a* of a function $g:[a,\infty )\rightarrow \mathbb{R}$ of order *α*, where $g^{(n)}(t)$ exists, is defined by

2.4 $$ \bigl(\mathbf{T}_{\alpha }^{a} g\bigr) (t)= \bigl(T_{\gamma }^{a} g^{(n)}\bigr) (t). $$

In case $a=0$, we write $\mathbf{T}_{\alpha }$.

Note that if $\alpha =n+1$, then $\gamma =1$ and the fractional derivative of *g* becomes $g^{(n+1)}(t)$. Also, when $n=0$ (or $\alpha \in (0,1)$), then $\gamma =\alpha $ and the definition coincides with that in Definition 2.1. From (2.4), it is an immediate consequence that if $n< \alpha \leq n+1$, then $\gamma =\alpha -n$ and if, moreover, the $(n+1)$st derivative (or the derivative of $g^{(n)}$) exists, then we have

2.5 $$ \bigl( \mathbf{T}_{\alpha }^{a} g\bigr) (t)= \bigl(T_{a}^{\gamma }g^{(n)}\bigr) (t)=(t-a)^{1- \gamma } g^{(n+1)}(t)=(t-a)^{1-\alpha +n}g^{(n+1)}(t). $$

### Lemma 2.3

([36])

*Assume that* $g:[a,\infty )\rightarrow \mathbb{R}$ *is continuous and* $0< \alpha \leq 1$. *Then*, *for all* $t>a$, *we have*

$$ T_{\alpha }^{a} I_{\alpha }^{a} g(t)=g(t). $$

In case of higher order, the following definition is valid.

### Definition 2.4

([37])

Let $\alpha \in (n,n+1]$ and set $\gamma = \alpha -n$. Then the left conformable fractional integral starting at *a* of order *α* is defined by

2.6 $$ \bigl(I_{\alpha }^{a} g \bigr) (t)= \textbf{I}_{n+1}^{a} \bigl((t-a)^{\gamma -1}g\bigr)= \frac{1}{n!} \int _{a}^{t} (t-x)^{n}(x-a)^{\gamma -1}g(x)\,\mathrm{d}x. $$

Notice that if $\alpha =n+1$ then $\gamma =1$ and hence

$$ \bigl(I_{\alpha }^{a} g\bigr) (t)=\bigl(\textbf{I}_{n+1}^{a} g\bigr) (t)=\frac{1}{n!} \int_{a} ^{t}(t-x)^{n} g(x)\,\mathrm{d}x, $$

which is the iterative integral of *g*, $n+1$ times over $(a,t]$.

Recalling that the left Riemann–Liouville fractional integral of order $\alpha >0$ starting from *a* is defined by

2.7 $$ \bigl(_{a}\textbf{I}^{\alpha }g\bigr) (t)= \frac{1}{\Gamma (\alpha )} \int_{a} ^{t}(t-s)^{\alpha -1} g(s)\,\mathrm{d}s, $$

we see that $(I_{\alpha }^{a} g)(t)= ({}_{a}\textbf{I}^{\alpha }g)(t)$ for $\alpha = n+1$, $n=0,1,2,\ldots $ .

### Example 2.5

In virtue of [37], we recall that

$$ \bigl({}_{a}\textbf{I}^{\alpha }(t-a)^{\mu -1}\bigr) (x)= \frac{\Gamma (\mu )}{\Gamma (\mu +\alpha )}(x-a)^{\alpha +\mu -1},\quad \alpha ,\mu >0. $$

Indeed, if $\mu \in \mathbb{R}$ such that $\alpha +\mu -n>0$, then the conformable fractional integral of $(t-a)^{\mu }$ of order $\alpha \in (n,n+1]$ is

2.8 $$ \bigl(I_{\alpha }^{a} (t-a)^{\mu }\bigr) (x)=\bigl(\textbf{I}_{n+1}^{a}(t-a)^{\mu +\alpha -n-1}\bigr) (x)= \frac{\Gamma (\alpha +\mu -n)}{\Gamma (\alpha +\mu +1)}(x-a)^{\alpha +\mu }. $$

The following is a generalization of Lemma 2.3.

### Lemma 2.6

([37])

*Assume that* $f:[a,\infty )\rightarrow \mathbb{R}$ *such that* $g^{(n)}(t)$ *is continuous and* $n< \alpha \leq n+1$. *Then*, *for all* $t>a$, *we have*

$$ \mathbf{T}_{\alpha }^{a} I_{\alpha }^{a} g(t)=g(t). $$

### Theorem 2.7

([37])

*Let* $\alpha \in (n,n+1] $ *and* $g:[a,\infty ) \rightarrow \mathbb{R}$ *be* $(n+1)$ *times differentiable for* $t>a$. *Then*, *for all* $t>a$, *we have*

2.9 $$ I_{\alpha }^{c} \mathbf{T}_{\alpha }^{a}(g) (t)= g(t)-\sum_{k=0}^{n} \frac{g ^{(k)}(a)(t-a)^{k}}{k!}. $$

### Example 2.8

In view of [37], we recall that the solution of the following conformable fractional initial value problem

2.10 $$ \bigl(T_{\alpha }^{a} x\bigr) (t)= \lambda x(t),\qquad x(a)=x_{0} $$

is

$$ x(t)=x_{0}e^{\lambda (t-a)^{\alpha }/\alpha }, \quad t>a, $$

for $\alpha \in (0,1]$.

## Results and discussion

Prior to proceeding to the main theorems, we state the following key lemma which was proved in [15].

### Lemma 3.1

([15])

*If* *A* *is positive and* *B*, *z* *are non*-*negative*, *then*

3.1 $$ Az^{2}-Bz^{\alpha }+(2-\alpha ) \alpha^{\alpha /(2-\alpha )}2^{2/( \alpha -2)} A^{-\alpha /(2-\alpha )} B^{2/(2-\alpha )}\geq 0 $$

*for any* $\alpha \in (0,2)$. *The equality holds if and only if* $B=z=0$.

The investigation of Lyapunov inequalities is delivered in two separate folds.

### A Lyapunov-type inequality for mixed forced conformable BVP

In this subsection, we consider the following mixed forced conformable BVP:

3.2 $$\begin{aligned} \textstyle\begin{cases} \mathbf{T}^{a}_{\alpha }x(t)+r_{1}(t)\vert x(t) \vert ^{\eta -1}x(t)+r_{2}(t)\vert x(t) \vert ^{ \delta -1}x(t)=g(t), \\ x(a)=x(b)=0, \end{cases}\displaystyle \end{aligned}$$

where $\alpha \in (1,2]$, the non-linearities satisfy (1.6), and the potential functions $r_{1}$, $r_{2}$, and forcing term *g* are real-valued integrable functions which do not require any sign restriction. The solution of (3.2) is valid for all real-valued functions $x(t)$ with $\mathbf{T}^{a}_{\alpha }x(t)$ exists and is integrable on $[a,b]$ such that it satisfies (3.2). The Lyapunov inequality for BVP (3.2) is established.

Consider the following (local) conformable BVP:

3.3 $$\begin{aligned} \textstyle\begin{cases} -(\mathbf{T}_{\alpha }^{c} x)(t)={\mathcal{F}}(t), \\ x(a)=x(b)=0 \end{cases}\displaystyle \end{aligned}$$

for $t\in (a,b)$, where $\alpha \in (1,2]$. The Green’s function and its properties are given in the following two lemmas.

#### Lemma 3.2

([44])

*x* *is a solution of BVP* (3.3) *if and only if it satisfies the integral equation*

3.4 $$ x(t)= \int_{a}^{b} H(t,s){\mathcal{F}}(s)\,\mathrm{d}s, $$

*where* *H* *is the Green’s function for BVP* (3.3) *defined by*

$$ H(t,s)=\frac{(t-a)(b-s)}{b-a}\,(s-a)^{ \alpha -2}- \textstyle\begin{cases} 0, & a \leq t \leq s\leq b, \\ (t-s)(s-a)^{\alpha -2}, & a \leq s \leq t\leq b. \end{cases} $$

#### Lemma 3.3

([44])

*The Green’s function* *H* *defined above has the following properties*:

(i) $H(t,s)\geq 0$ *for all* $a\leq t,s \leq b$,
(ii) $\max_{t \in [a,b]} H(t,s)=H(s,s)$ *for* $s \in [a,b]$,
(iii) $H(s,s)$ *has a unique maximum at* $s_{0}=[a+(\alpha -1)b]/ \alpha $, *and we have*
  $$ \max_{s\in [a,b]}H(s,s)=H(s_{0},s_{0})= \frac{(b-a)^{\alpha -1}(\alpha -1)^{\alpha -1}}{\alpha^{\alpha }}. $$

The following Lyapunov-type inequality was proved for the BVP:

3.5 $$\begin{aligned} \textstyle\begin{cases} (\mathbf{T}_{\alpha }^{a} x)(t)+r(t)x(t)=0, \\ x(a)=x(b)=0 \end{cases}\displaystyle \end{aligned}$$

in the frame of conformable derivatives [44].

#### Theorem 3.4

(Lyapunov-type inequality [44])

*Suppose that* *a*, *b*, $a>b$ *are consecutive zeros of a non*-*trivial solution of BVP* (3.5), *then the inequality*

3.6 $$ \int_{a}^{b}\bigl\vert r(t) \bigr\vert \,\mathrm{d}t>\frac{\alpha^{\alpha }}{(\alpha -1)^{ \alpha -1}(b-a)^{\alpha -1}} $$

*holds*.

#### Remark 3.5

If $\alpha =2$, then (3.6) reduces to the classical Lyapunov inequality (1.2).

In what follows, we make use of the following notation:

$$ u^{\pm }=\max \{\pm u,0\}. $$

#### Theorem 3.6

(Hartman-type inequality)

*If* $x(t)$ *is a positive solution of BVP* (3.2) *in* $(a,b)$, *then the inequality*

3.7 $$\begin{aligned} & \biggl( \int_{a}^{b}(b-t) (t-a)^{\alpha -1} \bigl[r_{1}^{+}(t)+r_{2}^{+}(t)\bigr]\,\mathrm{d}t \biggr) \\ &\qquad {} \times \biggl( \int_{a}^{b}(b-t) (t-a)^{\alpha -1}\bigl[ \eta_{0}r _{1} ^{+}(t)+\delta_{0}r_{2}^{+}(t)+g^{-}(t) \bigr]\,\mathrm{d}t \biggr) \\ &\quad {}> \frac{1}{4}(b-a)^{2} \end{aligned}$$

*holds*, *where* $\eta_{0}$ *and* $\delta_{0}$ *are defined in* (1.8).

#### Proof

Let *x* be a positive solution of (3.2) in $(a,b)$ with $x(a)=x(b)=0$. On the basis of Lemma 3.2, the solution of BVP (3.2) is given by

3.8 $$ x(t)= \int_{a}^{b} H(t,s) \bigl[ r_{1}(s) x^{\eta }(s)+r_{2}(s) x^{ \delta }(s)-g(s) \bigr]\,\mathrm{d}s $$

for any $t\in (a,b)$, where $H(t,s)$ is the Green’s function of BVP (3.3).

On the other hand, we have from (ii) of Lemma 3.3 that

3.9 $$\begin{aligned} 0&\leq H(t,s)\leq H(s,s) \\ &= \frac{(b-s)(s-a)^{\alpha -1}}{b-a}, \quad s \in (a,b). \end{aligned}$$

Let $x(t_{0})=\max_{t\in (a,b)}x(t)$. Then, by (3.8) and (3.9), we have

3.10 $$\begin{aligned} x(t_{0}) &= \int_{a}^{b} H(t_{0},s) \bigl[ r_{1}(s)x^{\eta }(s)+r_{2}(s)x ^{\delta }(s)-g(s) \bigr]\,\mathrm{d}s \\ &\leq \frac{1}{b-a} \int_{a}^{b} (b-s) (s-a)^{\alpha -1} \bigl[ r_{1} ^{+}(s)x^{\eta }(s)+r_{2}^{+}(s)x^{\delta }(s)+g^{-}(s) \bigr]\,\mathrm{d}s \\ &\leq \mu_{1} x^{\eta }(t_{0})+\mu_{2} x^{\delta }(t_{0})+\mu , \end{aligned}$$

where

3.11 $$\begin{aligned} \mu_{j}= \frac{1}{b-a} \int_{a}^{b} (b-s) (s-a)^{\alpha -1}r_{j}^{+} (s)\,\mathrm{d}s, \quad j=1,2 \end{aligned}$$

and

$$ \mu =\frac{1}{b-a} \int_{a}^{b} (b-s) (s-a)^{\alpha -1}g^{-}(s)\,\mathrm{d}s. $$

By the help of inequality (3.1) in Lemma 3.1 with $A=1$ and $B=1$, we reach the quadratic inequality

3.12 $$ (\mu_{1} +\mu_{2})x^{2}(t_{0})-x(t_{0})+ \eta_{0}\mu_{1}+\delta_{0}\mu_{2}+ \mu >0. $$

This is possible only if

$$ (\mu_{1}+\mu_{2}) (\eta_{0}\mu_{1}+ \delta_{0}\mu_{2}+\mu )>\frac{1}{4}. $$

This completes the proof. □

#### Theorem 3.7

(Hartman-type inequality)

*If* $x(t)$ *is a negative solution of BVP* (3.2) *in* $(a,b)$, *then the inequality*

3.13 $$\begin{aligned} & \biggl( \int_{a}^{b}(b-t) (t-a)^{\alpha -1} \bigl[r_{1}^{+}(t)+r_{2}^{+}(t)\bigr]\,\mathrm{d}t \biggr) \\ &\qquad {} \times \biggl( \int_{a}^{b}(b-t) (t-a)^{\alpha -1}\bigl[ \eta_{0}r _{1} ^{+}(t)+\delta_{0}r_{2}^{+}(t)+g^{+}(t) \bigr]\,\mathrm{d}t \biggr) \\ &\quad {}> \frac{1}{4}(b-a)^{2} \end{aligned}$$

*holds*, *where* $\eta_{0}$ *and* $\delta_{0}$ *are defined in* (1.8).

#### Proof

Let *x* be a negative solution of (3.2) in $(a,b)$ with $x(a)=x(b)=0$. In fact, if $x(t)<0$ for $t\in (a,b)$, then we can consider $z(t)=-x(t)$ as a positive solution of the BVP

3.14 $$\begin{aligned} \textstyle\begin{cases} \mathbf{T}^{a}_{\alpha }z(t)+r_{1}(t)\vert z(t) \vert ^{\eta -1}z(t)+r_{2}(t)\vert z(t) \vert ^{ \delta -1}z(t)=-g(t), \\ z(a)=z(b)=0 \end{cases}\displaystyle \end{aligned}$$

in $(a,b)$. By using (3.3) and (3.14), $z(t)$ can be expressed as

3.15 $$ z(t)= \int_{a}^{b}H(t,s) \bigl[ r_{1}(s)z^{\eta }(s)+r_{2}(s)z^{\delta }(s)+g(s) \bigr]\,\mathrm{d}s $$

for any $t\in (a,b)$, where $H(t,s)$ is the Green’s function of BVP (3.3).

Let $z(t_{*})=\max_{t\in (a,b)}z(t)$. Then, by (3.15) and (3.9), we have

$$\begin{aligned} z(t_{*}) &= \int_{a}^{b}H(t_{*},s) \bigl[ r_{1}(s)z^{\eta }(s)+r_{2}(s)z ^{\delta }(s)+g(s) \bigr]\,\mathrm{d}s \\ &\leq \frac{1}{b-a} \int_{a}^{b}(b-s) (s-a)^{\alpha -1} \bigl[ r_{1} ^{+}(s)z ^{\eta }(s)+r_{2}^{+}(s)z^{\delta }(s)+g^{+}(s) \bigr]\,\mathrm{d}s \\ &\leq \mu_{1}z^{\eta }(t_{*})+\mu_{2}z^{\delta }(t_{*})+ \nu , \end{aligned}$$

where $\mu_{1}$ and $\mu_{2}$ are defined in (3.11) and

$$ \nu =\frac{1}{b-a} \int_{a}^{b}(b-s) (s-a)^{\alpha -1}g^{+}(s)\,\mathrm{d}s. $$

Repeating the same steps as in Theorem 3.6, we obtain (3.13), which completes the proof. □

#### Theorem 3.8

(Hartman-type inequality)

*If* $x(t)$ *is a solution of BVP *(3.2) *which has no zero in* $(a,b)$, *then the inequality*

3.16 $$\begin{aligned} & \biggl( \int_{a}^{b}(b-t) (t-a)^{\alpha -1} \bigl[r_{1}^{+}(t)+r_{2}^{+}(t)\bigr]\,\mathrm{d}t \biggr) \\ &\qquad {} \times \biggl( \int_{a}^{b}(b-t) (t-a)^{\alpha -1}\bigl[ \eta_{0}r _{1} ^{+}(t)+\delta_{0}r_{2}^{+}(t)+ \bigl\vert g(t) \bigr\vert \bigr]\,\mathrm{d}t \biggr) \\ &\quad {}> \frac{1}{4}(b-a)^{2} \end{aligned}$$

*holds*, *where* $\eta_{0}$ *and* $\delta_{0}$ *are defined in* (1.8).

#### Proof

Let *x* be a solution of (3.2) which has no zero in $(a,b)$ with $x(a)=x(b)=0$. Since either $x(t)>0$ or $x(t)<0$ for $t\in (a,b)$ and $g^{\pm }(t)\leq \vert g(t) \vert $, by (3.7) and (3.13) we obtain (3.16). This completes the proof of Theorem 3.8. □

Upon employing

3.17 $$\begin{aligned} \max_{t\in (a,b)} \bigl\{ (b-t) (t-a)^{\alpha -1} \bigr\} =\frac{( \alpha -1)^{\alpha -1}}{\alpha^{\alpha }}(b-a)^{\alpha } \end{aligned}$$

for all $t\in (a,b)$, as inferred from (iii) of Lemma 3.3, we deduce that inequalities (3.7) and (3.16) in Theorem 3.6, Theorem 3.7, and Theorem 3.8 imply the following Lyapunov-type inequalities, respectively.

#### Theorem 3.9

(Lyapunov-type inequality)

*If* $x(t)$ *is a positive solution of BVP* (3.2) *in* $(a,b)$, *then the inequality*

$$\begin{aligned} & \biggl( \int_{a}^{b}\bigl[r_{1}^{+}(t)+r_{2}^{+}(t) \bigr]\,\mathrm{d}t \biggr) \biggl( \int_{a}^{b}\bigl[\eta_{0}r_{1}^{+}(t)+ \delta_{0}r_{2}^{+}(t)+g^{-}(t)\bigr]\,\mathrm{d}t \biggr) >\frac{\alpha_{0}}{(b-a)^{2\alpha -2}} \end{aligned}$$

*holds*, *where* $\eta_{0}$ *and* $\delta_{0}$ *are defined in* (1.8), *and*

3.18 $$\begin{aligned} \alpha_{0}=\frac{\alpha^{2\alpha }}{4(\alpha -1)^{2\alpha -2}}. \end{aligned}$$

#### Theorem 3.10

(Lyapunov-type inequality)

*If* $x(t)$ *is a negative solution of BVP* (3.2), *then the inequality*

$$\begin{aligned} & \biggl( \int_{a}^{b}\bigl[r_{1}^{+}(t)+r_{2}^{+}(t) \bigr]\,\mathrm{d}t \biggr) \biggl( \int_{a}^{b}\bigl[\eta_{0}r_{1}^{+}(t)+ \delta_{0}r_{2}^{+}(t)+g^{+}(t)\bigr]\,\mathrm{d}t \biggr) >\frac{\alpha_{0}}{(b-a)^{2\alpha -2}} \end{aligned}$$

*holds*, *where* $\eta_{0}$, $\delta_{0}$, *and* $\alpha_{0}$ *are defined in *(1.8) *and *(3.18), *respectively*.

#### Theorem 3.11

(Lyapunov-type inequality)

*If* $x(t)$ *is a solution of BVP* (3.2) *which has no zero in* $(a,b)$, *then the inequality*

3.19 $$\begin{aligned} & \biggl( \int_{a}^{b}\bigl[r_{1}^{+}(t)+r_{2}^{+}(t) \bigr]\,\mathrm{d}t \biggr) \biggl( \int_{a}^{b}\bigl[\eta_{0}r_{1}^{+}(t)+ \delta_{0}r_{2}^{+}(t)+\bigl\vert g(t) \bigr\vert \bigr] \,\mathrm{d}t \biggr) >\frac{\alpha_{0}}{(b-a)^{2\alpha -2}} \end{aligned}$$

*holds*, *where* $\eta_{0}$, $\delta_{0}$, *and* $\alpha_{0}$ *are defined in *(1.8) *and *(3.18), *respectively*.

If we set $r_{2}(t)=0$ in BVP (3.2), then we obtain the following particular forced sub-linear BVP:

3.20 $$\begin{aligned} \textstyle\begin{cases} \mathbf{T}^{a}_{\alpha }x(t)+r_{1}(t)\vert x(t) \vert ^{\eta -1}x(t)=g(t), \\ x(a)=x(b)=0. \end{cases}\displaystyle \end{aligned}$$

#### Theorem 3.12

*If* $x(t)$ *is solution of BVP* (3.20) *which has no zero in* $(a,b)$, *then the following Hartman*-*type and Lyapunov*-*type inequalities hold*:

(i) 3.21 $$\begin{aligned} & \biggl( \int_{a}^{b}(b-t) (t-a)^{\alpha -1}r_{1}^{+}(t)\,\mathrm{d}t \biggr) \\ &\qquad {} \times \biggl( \int_{a}^{b}(b-t) (t-a)^{\alpha -1}\bigl[ \eta_{0}r _{1} ^{+}(t)+\bigl\vert g(t) \bigr\vert \bigr]\,\mathrm{d}t \biggr) \\ &\quad {} >\frac{1}{4}(b-a)^{2}, \end{aligned}$$
(ii) $$\begin{aligned} & \biggl( \int_{a}^{b}r_{1}^{+}(t)\,\mathrm{d}t \biggr) \biggl( \int_{a} ^{b}\bigl[ \eta_{0}r_{1}^{+}(t)+ \bigl\vert g(t) \bigr\vert \bigr]\,\mathrm{d}t \biggr) >\frac{\alpha _{0}}{(b-a)^{2 \alpha -2}}, \end{aligned}$$

*where* $\eta_{0}$ *and* $\alpha_{0}$ *are defined in *(1.8) *and *(3.18), *respectively*.

If we set $r_{1}(t)=0$ in BVP (3.2), then we obtain the following particular forced super-linear BVP:

3.22 $$\begin{aligned} \textstyle\begin{cases} \mathbf{T}^{a}_{\alpha }x(t)+r_{2}(t)\vert x(t) \vert ^{\delta -1}x(t)=g(t), \\ x(a)=x(b)=0. \end{cases}\displaystyle \end{aligned}$$

#### Theorem 3.13

*If* $x(t)$ *is a solution of BVP* (3.22) *which has no zero in* $(a,b)$, *then the following Hartman*-*type and Lyapunov*-*type inequalities hold*:

(i) 3.23 $$\begin{aligned} & \biggl( \int_{a}^{b}(b-t) (t-a)^{\alpha -1}r_{2}^{+}(t)\,\mathrm{d}t \biggr) \\ &\qquad {} \times \biggl( \int_{a}^{b}(b-t) (t-a)^{\alpha -1}\bigl[ \delta_{0}r _{2}^{+}(t)+\bigl\vert g(t) \bigr\vert \bigr]\,\mathrm{d}t \biggr) \\ &\quad {}>\frac{1}{4}(b-a)^{2}, \end{aligned}$$
(ii) $$\begin{aligned} & \biggl( \int_{a}^{b}r_{2}^{+}(t)\,\mathrm{d}t \biggr) \biggl( \int_{a} ^{b}\bigl[ \delta_{0}r_{2}^{+}(t)+ \bigl\vert g(t) \bigr\vert \bigr]\,\mathrm{d}t \biggr) >\frac{\alpha _{0}}{(b-a)^{2 \alpha -2}}, \end{aligned}$$

*where* $\delta_{0}$ *and* $\alpha_{0}$ *are defined in *(1.8) *and *(3.18), *respectively*.

#### Remark 3.14

If $\alpha =2$, then Theorem 3.8 and Theorem 3.11 reduce to Theorem 2.3 and Theorem 2.4 in [16], respectively.

As $\eta \to 1^{-}$ and $\delta \rightarrow 1^{+}$, BVP (3.2) reduces to the forced problem

3.24 $$\begin{aligned} \textstyle\begin{cases} \mathbf{T}^{a}_{\alpha }x(t)+r(t)x(t)=g(t), \\ x(a)=x(b)=0, \end{cases}\displaystyle \end{aligned}$$

where $r(t)=r_{1}(t)+r_{2}(t)$. Furthermore, since $\lim_{\eta \to 1^{-}}\eta_{0}=\lim_{\delta \to 1^{+}}\delta_{0}=1/4 $ and in view of Theorem 3.8 and Theorem 3.11, the following corollary is an immediate consequence.

#### Corollary 3.15

*If* $x(t)$ *is a solution of BVP* (3.24) *which has no zero in* $(a,b)$, *then the following Hartman*-*type and Lyapunov*-*type inequalities hold*:

(i) $$\begin{aligned} &\biggl( \int_{a}^{b}(b-t) (t-a)^{\alpha -1}r^{+}(t)\,\mathrm{d}t \biggr) \biggl( \int_{a}^{b}(b-t) (t-a)^{\alpha -1} \bigl[r^{+}(t)+4\bigl\vert g(t) \bigr\vert \bigr]\,\mathrm{d}t \biggr) \\ &\quad >(b-a)^{2}, \end{aligned}$$
(ii) $$\begin{aligned} & \biggl( \int_{a}^{b}r^{+}(t)\,\mathrm{d}t \biggr) \biggl( \int_{a}^{b}\bigl[r ^{+}(t)+4\bigl\vert g(t) \bigr\vert \bigr] \,\mathrm{d}t \biggr) >\frac{4\alpha_{0}}{(b-a)^{2 \alpha -2}}, \end{aligned}$$

*where* $\alpha_{0}$ *is defined in *(3.18).

#### Remark 3.16

If we set $\alpha =2$ and $g(t)=0$ in (3.24), then inequalities (i) and (ii) in Corollary 3.15 reduce to the classical Hartman (1.4) and Lyapunov inequalities (1.3) for BVP (1.1). Furthermore, one can easily note that (ii) of Corollary 3.15 implies Theorem 3.4 by taking $g(t)=0$.

### A Lyapunov-type inequality for mixed forced sequential conformable BVP

In this section, we consider the following mixed forced sequential conformable BVP:

3.25 $$\begin{aligned} \textstyle\begin{cases} \mathbf{T}^{a}_{\alpha }\mathbf{T}^{a}_{\alpha }x(t) +r_{1}(t) \vert x(t) \vert ^{ \eta -1}x(t)+ r_{2}(t)\vert x(t) \vert ^{\delta -1}x(t)=g(t), \\ x(a)=x(b)=0, \end{cases}\displaystyle \end{aligned}$$

where $\alpha \in (1/2,1]$ and the non-linearities, the potential functions, and forcing term satisfy the same assumptions as in (3.2). The solution of (3.25) is valid for $x(t)$ defined on $[a,b]$ such that $\mathbf{T}^{a}_{\alpha }\mathbf{T}^{a}_{\alpha }x(t)$ exists and is integrable on $[a,b]$ and such that $x(t)$ satisfies (3.25). The Lyapunov inequality for (3.25) is established.

Consider the following sequential conformable BVP:

3.26 $$\begin{aligned} \textstyle\begin{cases} (\mathbf{T}_{\alpha }^{a} \circ \mathbf{T}_{\alpha }^{a} x)(t)={\mathcal{F}}(t), \\ x(a)=x(b)=0 \end{cases}\displaystyle \end{aligned}$$

for $t\in (a,b)$, where $\alpha \in (1/2,1]$. The following two lemmas, which were developed in [44], contain the Green’s function corresponding to BVP (3.26) and its estimate properties.

#### Lemma 3.17

*x* *is a solution of BVP *(3.26) *if and only if it satisfies the integral equation*

3.27 $$ x(t)= \int_{a}^{b} G(t,s){\mathcal{F}}(s)\,\mathrm{d}s, $$

*where* *G* *is the Green’s function of BVP *(3.26) *defined by*

$$ G(t,s)= \textstyle\begin{cases} g_{1}(t,s), & a \leq s \leq t\leq b, \\ g_{2}(t,s), & a \leq t \leq s\leq b \end{cases} $$

*such that*

$$ g_{1}(t,s)=\frac{1}{\alpha }(s-a)^{2\alpha -1} \biggl[ 1- \biggl( \frac{t-a}{b-a} \biggr) ^{\alpha } \biggr] $$

*and*

$$ g_{2}(t,s)=\frac{1}{\alpha }(s-a)^{\alpha -1}(t-a)^{\alpha } \biggl[ 1- \biggl( \frac{s-a}{b-a} \biggr) ^{\alpha } \biggr] . $$

#### Lemma 3.18

*The Green’s function* *G* *defined above has the following properties*:

(i) $G(t,s)\geq 0$ *for all* $a\leq t,s \leq b$,
(ii) $\max_{t \in [a,b]} G(t,s)=G(s,s)$ *for* $s \in [a,b]$,
(iii) $f(s)=G(s,s)$ *has a unique maximum at*
  3.28 $$\begin{aligned} s_{0}=\Lambda (a,b,\alpha ):=a+(b-a) (2\alpha -1)^{1/\alpha }(3\alpha -1)^{-1/\alpha }, \end{aligned}$$
  *and we have*
  3.29 $$\begin{aligned} \max_{s \in [a,b]} G(s,s)=G(s_{0},s_{0})=(2 \alpha -1)^{2-1/\alpha }(3 \alpha -1)^{1/\alpha -3}(b-a)^{2\alpha -1}. \end{aligned}$$

For the sequential BVP

3.30 $$\begin{aligned} \textstyle\begin{cases} (\mathbf{T}_{\alpha }^{a} \circ \mathbf{T}_{\alpha }^{a}x)(t)+r(t)x(t)=0, \\ x(a)=x(b)=0, \end{cases}\displaystyle \end{aligned}$$

Abdeljawad et al. [44] established the following Lyapunov-type inequality.

#### Theorem 3.19

(Lyapunov-type inequality)

*If* $x(t)$ *is a solution of BVP *(3.30) *which has no zero in* $(a,b)$, *then the inequality*

3.31 $$ \int_{a}^{b} \bigl\vert r(t) \bigr\vert \,\mathrm{d}t>\frac{1}{G (\Lambda (a,b,\alpha ), \Lambda (a,b,\alpha ) )}=\frac{(2\alpha -1)^{1/\alpha -2}(3\alpha -1)^{3-1/\alpha }}{(b-a)^{2\alpha -1}} $$

*holds*, *where* $\Lambda (a,b,\alpha )$ *is defined in *(3.28).

#### Remark 3.20

Since

$$ \lim_{\alpha \to 1^{-}}G \bigl(\Lambda (a,b,\alpha ),\Lambda (a,b,\alpha ) \bigr)=\frac{1}{4}(b-a), $$

the classical Lyapunov inequality (1.2) is obtained as $\alpha \rightarrow 1^{-}$. In this case, one may also deduce that $(T_{\alpha }^{a} \circ T_{\alpha }^{a}x)(t)\to x''(t)$ as $\alpha \rightarrow 1^{-}$.

#### Theorem 3.21

(Hartman-type inequality)

*If* $x(t)$ *is a solution of BVP *(3.25) *which has no zero in* $(a,b)$, *then the inequality*

3.32 $$\begin{aligned} \biggl( \int_{a}^{b}{\mathcal{U}}(t)\bigl[r_{1}^{+}(t)+r_{2}^{+}(t) \bigr]\,\mathrm{d}t \biggr) \biggl( \int_{a}^{b}{\mathcal{U}}(t)\bigl[ \eta_{0}r_{1}^{+}(t)+\delta_{0}r _{2}^{+}(t)+\bigl\vert g(t) \bigr\vert \bigr]\,\mathrm{d}t \biggr) >\frac{1}{4}\alpha^{2} \end{aligned}$$

*holds*, *where*

$$ \mathcal{U}(t)=(t-a)^{2\alpha -1} \biggl[ 1- \biggl( \frac{t-a}{b-a} \biggr) ^{\alpha } \biggr] , $$

*and that* $\eta_{0}$ *and* $\delta_{0}$ *are defined in *(1.8).

#### Proof

In the proof, we make use of the solution representation (3.27) of BVP (3.26) and the properties of the Green’s function $G(t,s)$ of BVP (3.26) given in Lemma 3.17 and (ii) of Lemma 3.18. Namely, we have that

$$ 0\leq G(t,s) \leq G(s,s)=\frac{1}{\alpha }\,{\mathcal{U}}(s), \quad s \in (a,b). $$

To avoid redundancy with the proof of Theorem 3.6, we omit the remaining part of the proof. □

#### Theorem 3.22

(Lyapunov-type inequality)

*If* $x(t)$ *is a solution of BVP *(3.25) *which has no zero in* $(a,b)$, *then the inequality*

3.33 $$\begin{aligned} \biggl( \int_{a}^{b}\bigl[r_{1}^{+}(t)+r_{2}^{+}(t) \bigr]\,\mathrm{d}t \biggr) \biggl( \int_{a}^{b}\bigl[\eta_{0}r_{1}^{+}(t)+ \delta_{0}r_{2}^{+}(t)+\bigl\vert g(t) \bigr\vert \bigr] \,\mathrm{d}t \biggr) >\frac{\alpha_{1}}{(b-a)^{4\alpha -2}} \end{aligned}$$

*holds*, *where*

$$ \alpha_{1}=\frac{1}{4}(3\alpha -1)^{6-2/\alpha }(2\alpha -1)^{2/ \alpha -4}, $$

*and that* $\eta_{0}$ *and* $\delta_{0}$ *are defined in *(1.8).

The proof is straightforward and it follows from (3.29) and inequality (3.32).

#### Remark 3.23

If $\alpha =1$ in Theorem 3.21 and Theorem 3.22, then inequalities (3.32) and (3.33) reduce to inequalities (3.16) and (3.19) in Theorem 3.8 and Theorem 3.11 with $\alpha =2$, respectively. Moreover, the limiting case $\eta \to 1^{-}$ and $\delta \rightarrow 1^{+}$ in Theorem 3.22 with $g(t)=0$ and $r(t)=r_{1}(t)+r_{2}(t)$ will imply Theorem 3.19.

Finally, we conclude this paper with the following remark. The results obtained for BVP (3.2) can be extended to the forced mixed non-linear problem in the frame of conformable derivatives with positive and negative coefficients

$$\begin{aligned} \textstyle\begin{cases} \mathbf{T}^{a}_{\alpha }x(t)\pm r_{1}(t)\vert x(t) \vert ^{\eta -1}x(t)\mp r_{2}(t)\vert x(t) \vert ^{ \delta -1}x(t)=g(t), \\ x(a)=x(b)=0. \end{cases}\displaystyle \end{aligned}$$

Moreover, similar results to Theorem 3.21 and Theorem 3.22 can be obtained for the sequential conformable problem with positive and negative coefficients of the form

$$\begin{aligned} \textstyle\begin{cases} \mathbf{T}^{a}_{\alpha }\mathbf{T}^{a}_{\alpha }x(t)\pm r_{1}(t)\vert x(t) \vert ^{ \eta -1}x(t)\mp r_{2}(t)\vert x(t) \vert ^{\delta -1}x(t)=g(t), \\ x(a)=x(b)=0. \end{cases}\displaystyle \end{aligned}$$

It might be of interest to find similar results for the more general equation of the form

$$\begin{aligned} \mathbf{T}^{a}_{\alpha }x(t)+\sum_{j=1}^{n}r_{j}(t) \bigl\vert x(t) \bigr\vert ^{\gamma_{j}-1}x(t)=g(t), \quad \alpha \in (1,2] \end{aligned}$$

or

$$\begin{aligned} \mathbf{T}^{a}_{\alpha }\mathbf{T}^{a}_{\alpha }x(t)+ \sum_{j=1}^{n}r _{j}(t)\bigl\vert x(t) \bigr\vert ^{\gamma_{j}-1}x(t)=g(t), \quad \alpha \in (1/2,1], \end{aligned}$$

where the non-linearities satisfy

$$ 0< \gamma_{1}< \cdots < \gamma_{m}< 1< \gamma_{m+1}< \cdots < \gamma_{n}< 2, $$

and no sign restriction is imposed on the forcing term $g(t)$ and the potential functions $r_{j}(t)$, $j=1,\ldots ,n$. The formulations and the proofs of the results are left to the reader.

## Conclusion

Conformable derivatives are naturally local fractional derivatives which allow deriving with respect to arbitrary order. Recently, it has been realized that conformable derivatives are essential in generating new types of fractional operators; see, for instance, the results reported in [39]. In this article, we have accommodated the concept and the properties of conformable derivatives to establish new generalized Lyapunov-type and Hartman-type inequalities for a boundary value problem with mixed non-linearities, $\alpha \in (1,2]$. In addition, the main results are carried out for the sequential conformable derivatives of the form $\mathbf{T}_{\alpha }^{a} \circ \mathbf{T}_{\alpha }^{a}$, $\alpha \in (1/2,1]$. The corresponding classical Lyapunov-type and Hartman-type inequalities are obtained in the limiting cases $\alpha \rightarrow 2^{-}$ and $\alpha \rightarrow 1^{-}$, respectively. The obtained inequalities generalize and compliment some existing results in the literature.
